# Testing the Efficacy of 2 Interventions to Improve Health Outcomes and Quality of Life Among Rural Older Adults Living With HIV: Protocol for a Randomized Controlled Trial

**DOI:** 10.2196/71429

**Published:** 2025-10-17

**Authors:** Andrew E Petroll, Sabina Hirshfield, Katherine G Quinn, Steven A John, Olivia H Algiers, Liam Randall, David Wyley Long, Timothy McAuliffe, Jennifer L Walsh

**Affiliations:** 1 Center for AIDS Intervention Research Department of Psychiatry and Behavioral Medicine The Medical College of Wisconsin Milwaukee, WI United States; 2 Division of Infectious Diseases Department of Medicine The Medical College of Wisconsin Milwaukee, WI United States; 3 STAR (Special Treatment and Research) Program Department of Medicine SUNY Downstate Health Sciences University New York, NY United States; 4 Institute for Sexual and Gender Health Department of Family Medicine and Community Health University of Minnesota Minneapolis United States; 5 Southern AIDS Coalition Powder Springs, GA United States

**Keywords:** HIV, older adults, rural, health outcomes, remotely delivered intervention

## Abstract

**Background:**

Rural people living with HIV in the United States have higher mortality rates and lower rates of HIV suppression compared to nonrural people living with HIV. In addition, compared to younger people living with HIV, older people living with HIV face numerous challenges to maintaining health and well-being. However, few interventions have targeted health or quality-of-life outcomes in rural older people living with HIV.

**Objective:**

This randomized controlled trial will evaluate the efficacy of 2 remotely delivered interventions—supportive-expressive peer social support groups and strengths-based case management—to improve viral suppression, medication adherence, quality of life, and depressive symptoms in people living with HIV aged >50 years in rural counties in the southern United States.

**Methods:**

We will enroll 352 rural older (aged >50 y) people living with HIV and test the interventions using a 2 (social support groups: yes or no) × 2 (strengths-based case management: yes or no) factorial design. Supportive-expressive peer social support groups aim to increase social support and lower HIV stigma, thereby improving HIV health outcomes and quality of life. Trained facilitators will deliver the 8 weekly sessions with a set curriculum to groups of 8 to 12 participants via videoconference. Strengths-based case management is an individual-level, individually tailored intervention delivered by trained staff. Over 5 sessions, the participant-staff duo selects a barrier impacting HIV care or quality of life and identifies short-term goals to overcome the barrier, focusing on the process of incremental problem-solving while recognizing accomplishments. We will assess HIV viral load—using participant-collected dried blood spot samples—at baseline and 8 and 12 months after the intervention. Using surveys, we will assess adherence, quality of life, depressive symptoms, and secondary outcomes at baseline and 4, 8, and 12 months after the intervention. We hypothesize that participants randomly assigned to each intervention will be more likely to be virally suppressed and adherent to antiretroviral therapy and have higher quality of life and fewer depressive symptoms at follow-up than those not assigned to each intervention. Secondary hypotheses are that, compared to those not receiving each intervention, participants in each intervention will report greater social support, self-efficacy, and likelihood of accessing needed services; less loneliness and internalized HIV stigma; and fewer structural barriers. Data will be analyzed using generalized linear mixed models.

**Results:**

Funded in April 2023, the study began enrollment in April 2024, with 177 participants having given consent by July 2025. Data collection will run through 2027 followed by analysis and publication by 2028.

**Conclusions:**

This study will evaluate 2 remotely delivered interventions for rural older people living with HIV for their effects on HIV health outcomes, quality of life, and depressive symptoms. If effective, these scalable interventions could improve outcomes for this growing population.

**Trial Registration:**

ClinicalTrials.gov NCT06269081; https://www.clinicaltrials.gov/study/NCT06269081

**International Registered Report Identifier (IRRID):**

DERR1-10.2196/71429

## Introduction

### Background

In the United States, more than 59,000 people living with HIV reside in rural areas, and approximately 2000 rural residents are newly diagnosed with HIV each year [1]. Rural people living with HIV have higher mortality rates than nonrural people living with HIV [2,3] and are more likely to have mental illness, substance dependence, and social isolation [4-8]. Despite having greater health care needs, rural people living with HIV are challenged by having health care providers with less experience in HIV care [4,9-11] and longer travel to medical specialists and other services [4,11-13]. Rural people living with HIV also fare worse on HIV-specific outcomes: they are less likely to be engaged in HIV care and to be virally suppressed than people living with HIV in urban areas [14].

Older adults living with HIV in rural areas face even greater challenges than their younger counterparts in overcoming barriers to care engagement and achieving viral suppression. Many older people living with HIV lack sufficient practical assistance and social support, and changes in physical and cognitive abilities may also impede care engagement and medication adherence [6,8,15,16]. To date, few interventions have been developed to improve health outcomes or quality of life among rural older people living with HIV. A 2021 systematic review of psychosocial interventions for older people living with HIV found only 9 such studies published since 2001, with the majority having fewer than 25 participants [17]. While most of these studies leveraged remotely delivered interventions, none were specifically focused on the challenges faced by rural older people living with HIV.

To address this research gap, our team has conducted a series of studies to understand challenges faced by rural older people living with HIV and develop intervention programs to improve their HIV health outcomes and quality of life. We first conducted qualitative interviews with 29 older people living with HIV in rural areas, finding that worries about HIV status disclosure, ageism, homonegativity, physical and psychological loneliness, and depression were commonly mentioned barriers to mental well-being and health care engagement [6].

We subsequently conducted a cross-sectional study of 446 rural older people living with HIV from throughout the United States and found that more than 25% had markers of low engagement in HIV care [18]. We identified a number of factors associated with low care engagement, including lower income, lack of internet access, higher stress, and longer travel distance to an HIV provider [18]. In addition, rural older people living with HIV with lower social support, greater experienced HIV stigma, lower quality of health care, experiences of discrimination in medical settings, and more structural barriers to care reported lower quality of life and poorer mental health [19]. Subsequent interviews with 27 personnel from organizations that provide medical or social services to rural older people living with HIV in the southern United States identified poverty, social isolation, stigma, and the lack of comprehensive case management services as barriers to care [20].

On the basis of this foundational research, we identified potential intervention targets to improve HIV health outcomes and quality of life, including social support, HIV stigma, structural barriers, and technology access. We then worked to adapt existing interventions and develop new interventions for remote delivery to this population. Four potential interventions were piloted with older rural people living with HIV to evaluate their feasibility, acceptability, and preliminary impact [21]. Two interventions—supportive-expressive peer social support groups and individual strengths-based case management (SBCM)—showed high levels of acceptability and positive preliminary impact [21].

### Supportive-Expressive Peer Social Support Group Intervention

Supportive-expressive peer social support groups are an evidence-based intervention with the objectives of increasing social support, reducing loneliness, and reducing internalized HIV stigma and in turn improving antiretroviral therapy (ART) adherence, viral suppression, depressive symptoms, and quality of life.

Social isolation is a prominent issue faced by many older people living with HIV, particularly those living in rural areas [12]. Low levels of perceived social support have been shown to negatively impact adherence to medication among older people living with HIV [22], and increasing social support can lead to improved health outcomes and reduced internalized HIV-related stigma [23]. A review of support groups for people living with HIV found that support group interventions were associated with reductions in morbidity and mortality and improvements in retention in care and quality of life [24]. However, the benefits of in-person support groups may be unavailable to rural-dwelling older people living with HIV due to their geographic isolation. Older rural people living with HIV may be unable to access these groups due to limited transportation options, long travel distances, and limited group availability in their areas.

On this evidence base, we lightly adapted the supportive-expressive peer group therapy intervention from Project Linkage [25] for remote delivery to older rural people living with HIV. When delivered by telephone, this intervention was shown to reduce depressive symptoms among older people living with HIV [25]. However, its efficacy for improving viral suppression, medication adherence, and quality of life for older people living with HIV has not been tested, nor has it been delivered by videoconference.

### SBCM Intervention

The SBCM model is derived from social action theory [26], which situates behavior as the consequence of individual-level factors (eg, attitudes and beliefs), environmental affordances, and structural barriers (eg, access to transportation or the lack thereof). SBCM is a protocol-driven, individually directed model of social support that focuses on identifying and leveraging individual strengths to address life stressors that function as obstacles to sustained behavior change (eg, HIV medication adherence). Core elements of the SBCM intervention include establishing rapport, strengths assessment, personal planning, resource acquisition, and interpersonal collaboration between the counselor and the participant. The SBCM model [27] has been successful in linking recently diagnosed or out-of-care people living with HIV to care and reducing HIV transmission risk among people living with HIV [28-31].

Given this evidence base, we selected and adapted an individually tailored SBCM intervention [21] for delivery by telephone or video to older people living with HIV with the goal of helping address the multiple structural barriers faced by rural older people living with HIV identified in our formative research [5,6]. SBCM is hypothesized to impact health outcomes through several pathways: first, by increasing levels of self-efficacy; second, by increasing the use of available services (eg, Supplemental Nutrition Assistance Program benefits, Medicare, transportation, or medication delivery services) through referrals and warm hand-offs to service agencies; third, by reducing structural barriers; and fourth, by increasing levels of perceived social support.

### Objectives and Aims

This 4-year study, supported by the National Institute of Nursing Research, will evaluate the efficacy of 2 interventions—supportive-expressive peer social support groups and SBCM—to improve viral suppression, medication adherence, quality of life, and depressive symptoms in a large sample of older (aged >50 y) people living with HIV who live in rural counties in the southern United States. Given the promise of these 2 distinct interventions, we opted to test both interventions simultaneously using an efficient 2×2 factorial design [32], which allows us to evaluate the main effect of each intervention as well as any interaction between the interventions. Group or individual interventions may better match the needs and capacity of community organizations wishing to implement interventions for rural older people living with HIV; therefore, demonstrating the efficacy of both interventions may maximize the potential for scale-up. We also chose to focus on multiple outcomes, including HIV health outcomes (viral suppression and ART adherence), broader quality of life, and mental health (depressive symptoms).

Here, we describe the protocol for the randomized controlled trial (RCT) evaluating these 2 interventions. We hypothesize that participants randomly assigned to each intervention will be more likely to be virally suppressed and adherent to HIV ART and will report higher quality of life and fewer depressive symptoms at follow-up than those not randomly assigned to each intervention. Secondary hypotheses are that, compared to those not randomly assigned to each intervention, participants randomly assigned to each intervention will report greater social support, self-efficacy, and likelihood of accessing needed services; less loneliness and internalized HIV stigma; and fewer structural barriers.

## Methods

### Overview of Study Design

**Figure 1:**
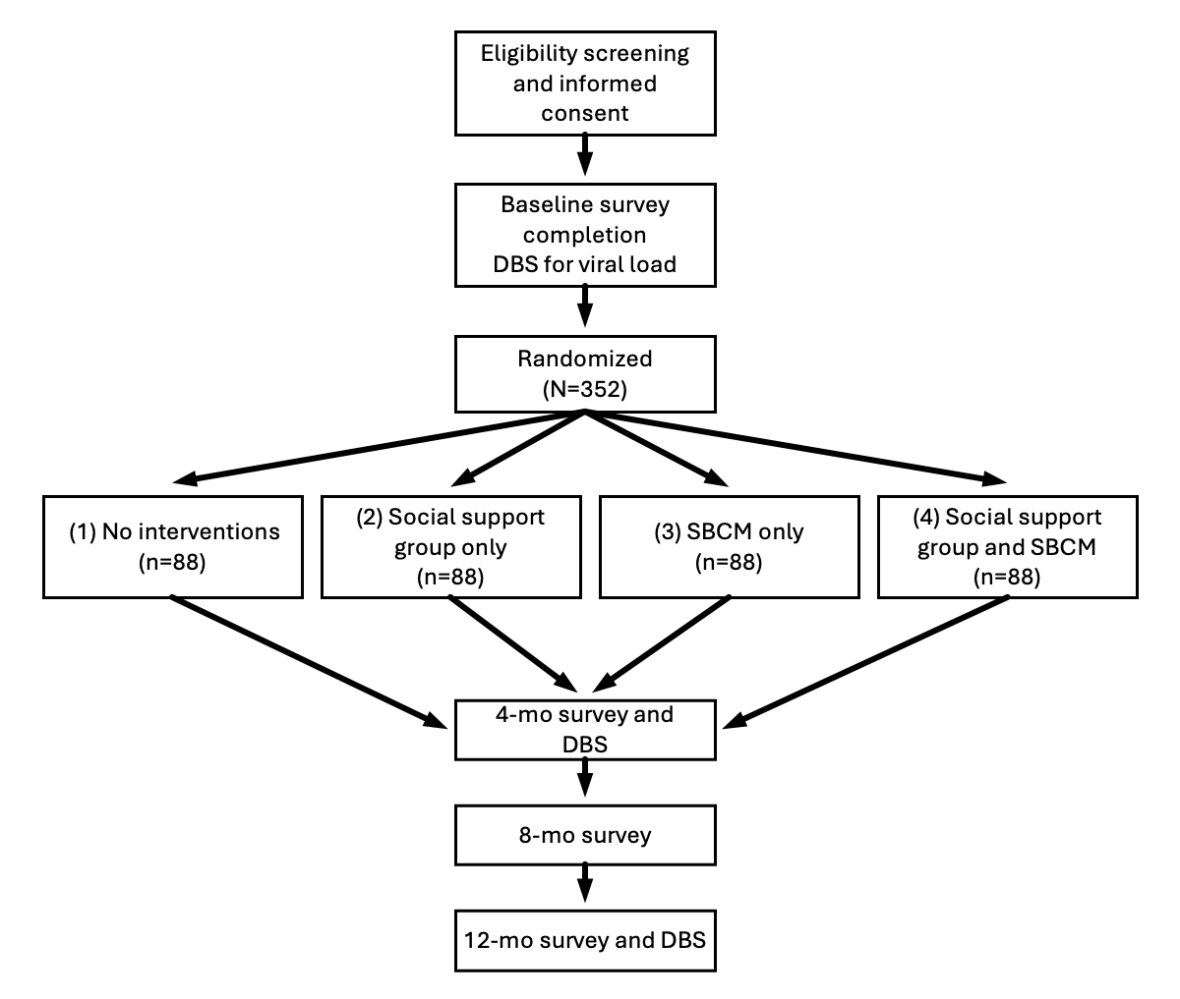
Participant flow through the study. DBS: dried blood spot; SBCM: strengths-based case management.

This RCT will assess the efficacy of 2 remotely delivered interventions—supportive-expressive peer social support groups and individual SBCM—for increasing viral suppression, ART adherence, and health-related quality of life, as well as decreasing depressive symptoms, among 352 rural older people living with HIV in the southern United States. We will assess these 2 interventions using a 2×2 factorial design. Interventions will be delivered over a 5- to 8-week period, and participants will complete surveys at baseline and 4-, 8-, and 12-month follow-ups and viral load assessments at baseline and 4 and 12 months. Participant flow through the study is shown in Figure 1. The study is being conducted by researchers at the Medical College of Wisconsin’s Center for AIDS Intervention Research and community partners at the Southern AIDS Coalition.

The study is registered on ClinicalTrials.gov (NCT06269081).

### Ethical Considerations

This study was reviewed and approved by the Medical College of Wisconsin Institutional Review Board (00044668). A waiver of documentation of written consent was obtained to reduce the risk of loss of confidentiality, particularly for participants completing the survey on paper by mail. Per the Code of Federal Regulations (45 CFR §46.117(c)) [33], written consent may be waived for research that involves minimal risk to participants and involves no procedures for which written consent is normally required outside of the research context. Participants enrolled in this study will still undergo an informed consent process with an informed consent document. All participants will speak with study staff, who will fully describe all elements of the study and answer participant questions. Participants completing the survey via the web will check a box indicating that they have read the informed consent document and agree to participate in the study; they will be emailed a copy of the informed consent document for their reference. Participants completing the study on paper will receive a copy of the informed consent document to read and keep for their reference; returning their surveys by mail will indicate consent to participate.

Participants will receive US $50 compensation for each survey completed and US $50 compensation for each dried blood spot (DBS) specimen returned. In addition, we will provide US $5 compensation for each core intervention session attended. Furthermore, participants can earn US $20 each for referring up to 2 additional participants to the study.

### Interventions

Both interventions tested in this RCT were previously shown to be feasible for remote delivery and acceptable to rural older people living with HIV during our pilot study [21].

#### Supportive-Expressive Peer Social Support Groups Intervention

This program, adapted from Project Linkage [25], is delivered to groups of approximately 8 to 12 participants by trained facilitators from the Southern AIDS Coalition. The groups meet weekly for 90-minute sessions over 8 weeks. Participants may choose to join each session by videoconference with or without video, or they may join by telephone for an audio-only experience. Each session has a unique focus: (1) introductions, program overview, and establishing group rules and expectations; (2) feelings around HIV diagnosis, stigma, and questions of “why did HIV happen to me?”; (3) values and priorities in life and thoughts about mortality; (4) uncertainty, challenges, and lack of control; (5) physician-patient relationship; (6) friend and family relationships; (7) body image and sense of self; and (8) reflection, summary, and closure. On the basis of participant feedback during our pilot, we incorporated the topic of HIV-related stigma into this intervention and also recruited group facilitators who had more shared lived experiences with participants. After the 8 group meetings, participants in groups are offered the opportunity to share their contact information with other participants if they are interested and sent instructions on how to host their own videoconference meetings should they wish to continue meeting to provide mutual social support.

#### SBCM Intervention

This adapted individually tailored SBCM intervention [21] is delivered by telephone or video by trained research staff over 5 sessions. The first session lasts 45 to 60 minutes and is focused on the following: (1) developing facilitator-participant rapport; (2) discussing participants’ background, current needs, and life challenges (eg, transportation, food insecurity, insurance, mental health, or technology); (3) identifying and ranking life challenges that participants experience as stressors and barriers to HIV care or quality of life; (4) identifying 1 stressor to focus on for the remaining sessions; (5) discussing past strategies to deal with the stressor; and (6) setting a short-term goal related to the stressor. After the first session, the facilitator compiles and sends the participant relevant, individually tailored resources (local and online) to aid in addressing contextual factors related to the stressor of focus. During the second session of similar length, the facilitator and participant will (1) review the resource list; (2) assess progress toward the short-term goal; (3) talk about accomplishments, strengths, and skills the participant has identified in their own life; and (4) revise the strategy to achieve the participant’s goals, incorporating identified strengths and skills to support progress. The final 3 sessions are optional, brief (15-30 min) check-ins, occurring at 1- to 2-week intervals, as determined by the participant and facilitator. These sessions focus on the achievement of an intermediate or long-term goal via the same collaborative process carried out in the first 2 sessions.

#### Control Condition: Information About Successfully Aging With HIV

All enrolled participants will be sent by US mail a printed packet of information on HIV treatment and health challenges, aging with HIV, and social support. The printed packet also includes an extensive list of resources available nationally and online for help with HIV information and support, mental health, violence, substance abuse, housing, transportation, finance, nutrition, health, and aging. Resources have been compiled from publicly available websites and brochures from the Centers for Disease Control and Prevention, National Institutes of Health, and HIV advocacy organizations [34,35]. This information will be the only intervention received by the 25% of participants not assigned to either active intervention.

### Participants

The target enrollment is 352 participants who meet the following eligibility criteria: (1) living with HIV, (2) aged more than 50 years, (3) living in 1 of the 17 target states (Textbox 1; target states include rural states prioritized by the “Ending the HIV Epidemic” initiative [36] as well as other states in the US Census southern region [37] that contain rural counties), (4) living in a county with an index of relative rurality of ≥0.4 [38], (5) having a telephone at home, (6) conversant in English, and (7) able to provide informed consent. We aim to recruit a sample that represents the population of rural people living with HIV in the southern United States with respect to sex, race, and ethnicity. Accordingly, we aim to have a sample that is comprised of at least 25% female participants and 50% racial and ethnic minority participants.

**Eligible states.**

Alabama, Arkansas, Delaware, Florida, Georgia, Kentucky, Louisiana, Maryland, Mississippi, Missouri, North Carolina, Oklahoma, South Carolina, Tennessee, Texas, Virginia, West Virginia

### Recruitment

Participants will be recruited in several ways. First, study information will be distributed to AIDS service organizations and other relevant community agencies serving people living with HIV in the 17 target states. Southern AIDS Coalition staff will assist by encouraging recruitment by agencies with whom they have ongoing relationships. These agencies will be asked to distribute study flyers to their clients or patients by US Postal Service, by email, or on social media. Second, we will recruit via social media, particularly Facebook (Meta). A Facebook page has been created for the study, and paid ads targeted to older people in the target counties will be posted. Posts on the study Facebook page may also be shared by AIDS service organizations, other community organizations, or individuals. In addition, other web- and mobile app–based recruitment methods may be used to reach potential participants, such as advertisements on dating apps used by sexual minority men in rural areas (eg, Growlr, Grindr, and SilverDaddies). Third, we will contact potential participants interested in research opportunities from research registries. Finally, we will invite participants to recruit friends or acquaintances who are people living with HIV to enroll. These approaches were used to successfully reach and enroll older rural people living with HIV in our previous studies [18,19,21].

### Procedures

Participants will be screened for eligibility using a web-based screener or by telephone. Study flyers contain the study web page, QR code, and our study telephone number. Online advertisements and postings contain a direct link to the screener. Study staff will contact eligible participants by telephone to describe the study, review the main components of the consent, and answer questions. Participants interested in enrolling will be sent either (1) a link to the web-based informed consent document and baseline survey by email or SMS text message or (2) a packet containing the informed consent document, baseline survey, and a stamped and addressed envelope for survey return via US mail. All participants interested in enrolling will also be sent a DBS study kit via US mail (refer to the HIV Viral Load Testing subsection). To guard against duplicate and fraudulent enrollments, we will require a unique and valid telephone number and postal address for each participant and speak with each participant before enrollment.

Only eligible participants who enroll in the study, complete a baseline survey, and return the baseline DBS study kit to the laboratory will be randomly assigned (refer to the Randomization subsection). After assignment, participants will be offered 0, 1, or 2 remote interventions depending on group (refer to the Interventions subsection). These interventions will be delivered over 5 to 8 weeks. Participants will complete follow-up assessments at 4, 8, and 12 months after assignment and provide DBS specimens at 4 and 12 months after assignment.

We will maximize retention by collecting multiple types of contact information (eg, telephone numbers, email address, and postal address) from participants upon enrollment. We will maximize participation in intervention sessions by providing reminders before each session using each participant’s preferred method of communication and by providing a modest compensation for each core intervention session attended.

### Quality Control and Fidelity Monitoring

Both interventions are manualized, and staff who deliver the interventions will be trained on using the manuals. Those delivering interventions will be equipped with checklists to further assist in obtaining fidelity. Sessions will be audio recorded (with participant agreement) to allow fidelity checks. Approximately 10% of the sessions will be randomly selected for formal review. These fidelity methods have been used successfully in our pilot [21].

### Randomization

Participants will be randomly assigned to 1 of 4 conditions in a 2 (social support groups: yes or no) × 2 (SBCM: yes or no) between-participants factorial design. Randomization will occur on an individual basis using the REDCap (Research Electronic Data Capture; Vanderbilt University) randomization module. An equal number of participants will be randomly assigned to each condition. Randomization will occur in blocks of 8 and be stratified based on sex. Neither participants nor study staff will be blinded to condition assignment.

### Measures

Primary and secondary outcome measures along with covariates are described in Table 1. Primary outcomes include DBS-assessed viral suppression, ART adherence, health-related quality of life, and depressive symptoms. The secondary outcomes are potential mediators of intervention effects, including social support, loneliness, internalized HIV stigma, general self-efficacy, treatment adherence self-efficacy, social and medical service use, and structural barriers (such as transportation as a barrier to care and housing instability). All primary and secondary outcomes are assessed in each survey (at baseline and 4, 8, and 12 mo) except for viral suppression, which is assessed via DBS at baseline and 4 and 12 months. Covariates are variables that will be controlled for in outcome analyses should they differ across randomized conditions or be associated with retention. We also include measures of feasibility and acceptability in the 4-month survey.

**Table 1:** Study measures.

Domains and subdomains			Description or scale
**Primary outcomes (baseline and 4, 8, and 12 mo)**			
	Viral suppression	Measured viral load <839 copies/mL in HemaSpot (Spot On Sciences) DBS^a^ sample (baseline and 4 and 12 mo only)	
	ART^b^ adherence	Wilson adherence scale [39] scores recoded to indicate imperfect vs perfect adherence, as suggested in the literature [40]	
	Quality of life	Scale score (0-100) on the WHOQOL-HIV BREF^c^ [41], calculated as in the study by Walsh et al [21]	
	Depressive symptoms	Scale score (0-27) on the PHQ-9^d^ depression module [42]	
**Secondary outcomes (baseline and 4, 8, and 12 mo)**			
	Social support	Scale score (1-5) on the MOS^e^ social support survey [43]	
	Loneliness	Scale score (1-5) on the loneliness survey from the NIH^f^ Toolbox [44]	
	Internalized HIV stigma	Scale score (1-5) on the internalized stigma subscale of the HIV Stigma Mechanisms Scale [45]	
	General self-efficacy	Scale score (1-5) on the New General Self-Efficacy Scale [46]	
	Treatment adherence self-efficacy	Scale score (0-4) on the HIV-ASES^g^ [47]	
	Service use	Participants will report which of 12 HIV-related social and medical services they have needed during the previous 3 mo and which of these needed services they were able to obtain [48]; a composite variable will indicate whether participants successfully accessed at least 1 needed service (0=no, 1=yes)	
	Structural barriers	Participants will report barriers faced in the past 3 mo, with items assessing the following: not having current health insurance, transportation as a barrier to health care, finances as a barrier to health care, not having enough food to eat, not having stable housing, and lacking eHealth literacy; the number of barriers will be summed (scores 0-6)	
**Covariates (baseline)**			
	Demographics, socioeconomic position, and health	Age, sex, gender identity, sexual orientation, race and ethnicity, education, income, employment, insurance status, years living with HIV, number of comorbid health conditions, and mode of survey completion (web-based vs paper)	
	Geography	State of residence and Index of Relative Rurality [38,49]	
	Use of other services	Use of HIV case management and participation in other social support groups	
**Acceptability and feasibility (4 mo)**			
	Acceptability of interventions	Satisfaction with interventions [50,51]	
	Acceptability of DBS testing	HemaSpot usability and acceptability questions [52]	
	Session attendance	Tracked by study staff	

^a^DBS: dried blood spot.

^b^ART: antiretroviral therapy.

^c^WHOQOL-HIV BREF: World Health Organization Quality of Life–HIV questionnaire short version.

^d^PHQ-9: Patient Health Questionnaire-9.

^e^MOS: Medical Outcomes Study.

^f^NIH: National Institutes of Health.

^g^HIV-ASES: HIV Treatment Adherence Self-Efficacy Scale.

### HIV Viral Load Testing

At enrollment, 4 months, and 12 months, participants will be sent a DBS kit by US Postal Service to self-collect a blood sample for HIV viral load testing. The DBS kits contain a HemaSpot collection device (volume capacity 80 μL); alcohol pads; 2 single-use retractable safety lancets; gauze pads; bandages; a biohazard bag labeled with participant ID; a postage-paid envelope for specimen return; and an instruwction card, written at an eighth-grade reading level, that describes how to collect the specimen. Participants are instructed to call study staff if they need additional assistance while collecting their samples. Participants will return specimens directly to the clinical laboratory for testing using a preaddressed shipping envelope. Shipping will be tracked by the study team, and specimen arrival will be logged by the laboratory.

HemaSpot devices with sufficient blood volume will be tested using the automated Abbott m2000 platform and the RealTime HIV-1 DBS reverse transcription polymerase chain reaction assay [53]. The assay has a limit of detection (LOD) of 2.92 log copies/mL (839 copies/mL) and can quantify up to 7.0 log copies/mL [54]. When no HIV-1 RNA is detected, the laboratory will report a result of “not detected” or “undetectable.” Detectable specimens will be binarized into qualitative and quantitative results based on the LOD. Qualitative viral load results will include those detectable but not quantifiable, as they fall below the LOD of 839 copies/mL. The laboratory will report quantitative viral load results when HIV-1 RNA is both detectable and quantifiable (≥839 copies/mL). The primary outcome of viral suppression will be defined as a viral load of <839 copies/mL.

Self-collection of DBS specimens for viral load monitoring was shown to be highly feasible and acceptable in our pilot work with older rural people living with HIV [21].

### Sample Size

With the recruitment of a sample size of 352 participants and anticipated 80% retention over time, tests for effects of the interventions will have a power of 0.80 or greater for a type I error of 0.05 to detect a difference of 0.35 SDs in the mean quality of life and depressive symptoms and of 35% in ART adherence and viral suppression between participants receiving versus not receiving each intervention at each follow-up assessment. A higher retention rate will increase power to detect smaller effects.

### Statistical Analysis

We will describe demographic characteristics for the sample and baseline levels of all primary and secondary outcomes. We will use ANOVA and chi-square tests to assess baseline differences in demographics and outcomes for those assigned to the 4 conditions. If significant differences between conditions exist, these will be accounted for in the primary analyses. Finally, we will assess possible sample attrition bias by examining demographic characteristics associated with loss to follow-up and by comparing retention across the 4 conditions.

We will use an intent-to-treat approach when analyzing outcomes [55]. The focus will be on the main effects of each intervention. We will use generalized linear mixed models (GLMMs) to test the impact of each intervention on the primary outcomes of viral suppression, ART adherence, quality of life, and depressive symptoms over time. This approach will test hypotheses within a repeated measures framework, with an individual identifier included in the models as a random effect to account for the correlation between participants’ outcomes over time. We will test hypotheses that the social support intervention and the SBCM intervention increase the likelihood of viral suppression and ART adherence, improve quality of life, and reduce depressive symptoms at 4-, 8-, and 12-month follow-ups, compared to not receiving each intervention, by examining the main effect of each intervention. The impact on viral suppression will be evaluated at 4 and 12 months only. GLMMs will also test the 2-way interaction between the interventions to understand whether the interventions are enhanced or offset by one another. If any differences between the randomized conditions emerge in preliminary analyses, covariates will be included in the models to account for these differences; similarly, if any covariates are associated with retention in the study, these covariates will be included in the models to improve the handling of missing data. Separate GLMMs will evaluate each hypothesis, with appropriate link functions used for binary outcomes (viral suppression and adherence) and continuous outcomes (quality of life and depressive symptoms). Hypotheses will be tested at the *P*<.05 level.

The same approach will be used to test the impact of each intervention on the secondary outcomes of social support, loneliness, stigma, self-efficacy, service use, and structural barriers. We will test hypotheses that the social support intervention and the SBCM intervention increase social support, self-efficacy, and uptake of needed services as well as decrease loneliness, stigma, and structural barriers at 4-, 8-, and 12-month follow-ups, compared to not receiving each intervention, by examining the main effect of each intervention.

Exploratory analyses will assess whether intervention effects differ based on demographic characteristics—including sex, race, and age (age 50-64 y vs age >65 y)—and baseline engagement in care (low vs high). Moderation analyses will shed light on who most benefits from the intervention programs, which can help guide better targeting of the programs or future efforts to refine and tailor interventions to meet the needs of any who do not benefit. We will test moderation by introducing interaction terms into the GLMM models assessing primary and secondary outcomes. In separate models for each moderator and outcome, we will include interactions between the moderator and assignment to each intervention as predictors of outcomes. Significant interaction terms will indicate differing intervention effects across subgroups. Significant interaction terms will be “plotted and probed” to examine intervention effects within different subgroups of participants [56].

Additional exploratory analyses will test whether the amount of each intervention received (ie, the number of sessions attended) or the mode of intervention delivery (ie, Zoom [Zoom Video Communications, Inc] vs telephone) relate to outcomes. These analyses will include only those participants randomly assigned to receive each intervention. GLMM models will examine associations between the number of intervention sessions attended or mode of intervention delivery (predictors) and outcomes (viral suppression, ART adherence, quality of life, and depressive symptoms), controlling for demographic covariates as needed. Separate models will estimate dose-response and mode-response relationships for each intervention-outcome combination.

Finally, analyses will explore the mediators of intervention effects on outcomes to shed light on the potential mechanisms of intervention effects. Understanding mediating mechanisms can help guide future efforts to optimize programs by capitalizing on the key processes involved in generating health outcomes. Mediation will be tested in a structural equation model, which will be fit in Mplus (Muthén & Muthén) [57] using a full-information maximum likelihood estimator robust to nonnormality. The model will include effect-coded interventions as predictors; social support, loneliness, self-efficacy, service use, and structural barriers as mediators; and viral suppression, adherence, quality of life, and depressive symptoms as outcomes. For a longitudinal test of mediation, models will include mediators assessed at 4 months and outcomes assessed at 8 and 12 months. Paths will lead from interventions to all mediators and from all mediators to all outcomes. Residual correlations between different mediators and different outcomes will be allowed, and covariates will be included as needed. Although we anticipate specific mechanisms for each intervention, this method will allow for a complete understanding of the mechanisms. We will use bootstrapped CIs to assess the indirect effects of the interventions on outcomes via mediators.

To inform future intervention implementation, we will also summarize information related to the acceptability and feasibility of each intervention and of the HemaSpot DBS testing for viral load. Acceptability will be assessed by survey items (Table 1). In terms of feasibility, we will summarize participation in each intervention (eg, number of sessions attended).

## Results

Recruitment and enrollment began in April 2024, with 177 participants consented as of July 2025. Recruitment is planned to conclude by April 2026. Final participant follow-ups will end by April 2027, followed by data analysis. RCT results will be disseminated by 2028 via peer-reviewed publications, presentations at scientific meetings, and ClinicalTrials.gov. Results will also be disseminated to AIDS service organizations and other community organizations via a written report and webinars. If the interventions are shown to be efficacious, full manuals will be made available to the public on our website.

## Discussion

### Contributions to the Field

This manuscript describes the protocol for an RCT testing the efficacy of 2 remotely delivered interventions that aim to improve viral suppression, medication adherence, health-related quality of life, and depressive symptoms among rural older people living with HIV in the southern United States. These interventions are designed to reach and engage rural older people living with HIV, a growing yet underserved population in need of health-related services. Innovations of this research include the focus on rural older people living with HIV; remote delivery of interventions to participants at home, overcoming transportation and distance barriers often faced by rural older people living with HIV; and objective assessment of viral suppression via self-collected DBS specimens.

### Limitations

Our study protocol has several limitations. First, although our multiple recruitment methods are designed to reach people living with HIV with varying levels of care engagement, including those with low engagement in care who are not virally suppressed, our partial reliance on AIDS service organizations for recruitment may lead to underrepresentation of those with the lowest care engagement. However, we have reached participants with suboptimal engagement in care and medication adherence and detectable viral loads in our previous studies with this population [18,19,21]. Similarly, because there may be lower barriers to screening online (vs placing a call to study staff) and because some recruitment activities will take place online, we may be more likely to reach participants who use the internet. To avoid excluding participants without internet access, we have ensured that there are offline pathways to recruitment (study flyers mailed to potential participants and displayed in community agencies), enrollment (via telephone), survey completion (on paper via mail), and intervention participation (via telephone). Finally, although our study uses an objective measure of viral suppression that has been successfully used in our pilot work, other outcome measures are self-reported.

### Conclusions

If successful, these manualized interventions, delivered to older rural people living with HIV at home, can be scaled up for rural settings across the United States, improving health equity. We hope that the results from this study will provide us with tools to improve health outcomes for a vastly understudied population and therefore advance the US “Ending the HIV Epidemic” initiative.

## Appendix

Multimedia Appendix 1 Peer review report by PPAH - Population and Public Health Approaches to HIV/AIDS Study Section, Healthcare Delivery and Methodologies Integrated Review Group (National Institutes of Health, USA).

## Abbreviations

ART: antiretroviral therapy
DBS: dried blood spot
GLMM: generalized linear mixed model
LOD: limit of detection
RCT: randomized controlled trial
REDCap: Research Electronic Data Capture
SBCM: strengths-based case management

## Data Availability

Upon study completion, the datasets generated or analyzed during this study will be available from the corresponding author on reasonable request.
