# Supplementary material for: Testing the Efficacy of 2 Interventions to Improve Health Outcomes and Quality of Life Among Rural Older Adults Living With HIV: Protocol for a Randomized Controlled Trial
Source: JMIR Res Protoc. 2025 Oct 17;14:e71429. doi: 10.2196/71429 (PMC12579288; doi:10.2196/71429)
Supplement: Multimedia Appendix 1 [file resprot_v14i1e71429_app1.pdf]

SUMMARY STATEMENT

(Privileged Communication)

PROGRAM CONTACT:
Shalanda Bynum
301-755-4355
bynumsa@csr.nih.gov

Release Date: 07/29/2022
Revised Date:

Application Number: 1 R01 NR020770-01

Principal Investigators (Listed Alphabetically):

PETROLL, ANDREW E
WALSH, JENNIFER LYNN (Contact)

Applicant Organization: MEDICAL COLLEGE OF WISCONSIN

Review Group: PPAH
Population and Public Health Approaches to HIV/AIDS Study Section
AIDS - EXP. REV.

Meeting Date: 07/14/2022
Council: OCT 2022
Requested Start: 12/01/2022
RFA/PA: PA20-183
PCC: CSDSB

Project Title: Testing the Efficacy of Two Interventions to Improve Health Outcomes and Quality of Life among Rural Older Adults Living with HIV
SRG Action: Impact Score:24 Percentile:10
Next Steps: Visit https://grants.nih.gov/grants/next_steps.htm
Human Subjects: 30-Human subjects involved - Certified, no SRG concerns
Animal Subjects: 10-No live vertebrate animals involved for competing appl.
Gender: 1A-Both genders, scientifically acceptable
Minority: 1A-Minorities and non-minorities, scientifically acceptable
Age: 3A-No children included, scientifically acceptable

Project Year	Direct Costs Requested	Estimated Total Cost
1	405,941	637,975
2	499,661	785,265
3	474,275	745,369
4	422,119	663,400
TOTAL	1,801,996	2,832,009

ADMINISTRATIVE BUDGET NOTE: The budget shown is the requested budget and has not been adjusted to reflect any recommendations made by reviewers. If an award is planned, the costs will be calculated by Institute grants management staff based on the recommendations outlined below in the COMMITTEE BUDGET RECOMMENDATIONS section.

1R01NR020770-01 WALSH, JENNIFER

RESUME AND SUMMARY OF DISCUSSION: Older persons living with HIV in Southern rural areas (OPLH) of the US are more likely than their urban counterparts to have been diagnosed at a more advanced stage of the disease, are less likely to initiate treatment and enroll in care, less likely to be retained in care, and more likely to suffer from various co-morbidities and to die. These applicants wish to evaluate the efficacy of two remotely delivered interventions: a supportive-expressive peer social support groups and strengths-based case management, which were found in a pilot study to be feasible, acceptable, and to show preliminary efficacy. The vulnerability of OPLH in rural areas renders this project significant as it is likely to exert an important public health impact for this population. The applicants form a strong team with the complementary skills to conduct this research. The MPI plan they have presented is justifiable, and their preliminary data support this research. The interventions are innovative in their being offered remotely in consideration of the lack of access to care rural OLPH are subject to; they will also be augmented by phone to allow for flexibility. The research design is robust: statistical methods proposed are strong; measures map on nicely to the constructs they are testing; the interventions are manualized, and thus, will promote fidelity which will be strongly monitored by the team. Moreover, in addition to self-report, viral load will be assessed using biologic measures. Some minor to moderate weaknesses were observed, however, that slightly reduced enthusiasm for this very strong application. These included the following: the applicants do not address the research impact of the different modes of delivery of the intervention (i.e., telephone vs remotely) and how it may influence the research; the pilot data provided only partial support for this project, namely the impact of the intervention on health-related quality of life; however, the intervention did not find effects on structural barriers, including lack of internet access; the success of the strengths-based case-management will rely on the availability of resources, but services are lacking or located far from them. Finally, the team may encounter difficulties in recruitment - contingency plans are not described in that regard nor are ways to maintain retention. Despite these observations, the greater majority of the committee assessed the potential impact of this application as ranging from high to extremely high, and a handful of reviewers assessed it as moderately strong.

DESCRIPTION (provided by applicant): More than 55,000 people living with HIV (PLH) in the US live in rural areas, and more than 2,300 rural residents are diagnosed with HIV each year. PLH who live in rural areas have higher mortality rates compared with non-rural PLH. Rural PLH are diagnosed with HIV at a more advanced stage than non-rural individuals and present for medical care later, making them more likely to face comorbidities and need complex medical care. Rural PLH are also less likely than their urban counterparts to remain engaged in HIV care and to be virally suppressed. Compared with younger PLH, older PLH may face additional challenges to maintaining their health and wellbeing, and older PLH who also live in rural areas face the doubly challenging prospect of maintaining adherence to HIV care and managing medical conditions while living in a rural environment. Few interventions aimed at increasing viral suppression and improving health-related quality of life (HRQOL) exist for rural older PLH. Our previous qualitative and survey research with rural older PLH nationwide (N = 476) identified low social support, HIV-related stigma, self-efficacy, and structural barriers (such as difficulties with housing, food access, transportation, and insurance) as key predictors of engagement in HIV care, viral suppression, and HRQOL for this population. Based on this, we previously piloted two remotely-delivered interventions for rural older PLH: supportive-expressive peer social support groups and strengths-based case management. The pilot with older PLH in the rural Southern U.S. found the interventions to be feasible, acceptable, and to show evidence of preliminary impact. Based on this work, we propose a full-scale trial to evaluate the efficacy of these two interventions. We will recruit 352 rural older PLH in the Southern U.S.— including in the states prioritized in the US HHS' "Ending the HIV Epidemic" (EtHE) plan—through partnerships with community agencies and online advertisements.

Following baseline surveys (completed online, by mail, or by phone) and HIV viral load testing (via self-collected dried blood spot samples), participants will be randomized to receive or not receive each intervention in a 2x2 factorial design. Follow-up surveys will occur at 4, 8, and 12 months, and viral load testing at 4 and 12 months. Surveys will assess medication adherence, depressive symptoms, HRQOL, covariates, and potential mediators (e.g., social support, HIV stigma, self- efficacy, structural barriers). Primary outcomes are viral suppression, antiretroviral therapy adherence, depressive symptoms and HRQOL, and secondary outcomes are potential mediating mechanisms. We hypothesize that both interventions will increase the proportion of participants that have viral suppression, levels of antiretroviral therapy adherence, and HRQOL and decrease depressive symptoms. Exploratory analyses will evaluate mediators and moderators of intervention effects. We will also assess the acceptability, feasibility, and costs of intervention delivery. Results from this study will provide us with tools to improve health outcomes for rural older PLH and to advance the EtHE plan to eliminate HIV transmission in the U.S.

PUBLIC HEALTH RELEVANCE: Engagement in HIV medical care and adherence to HIV medications are both essential in improving health outcomes among people living with HIV (PLH), but PLH living in rural areas—who suffer higher mortality rates than their urban counterparts—can confront multiple barriers to care engagement and adherence, especially as they face the logistical, medical, and social challenges associated with aging. This project will test the efficacy of two interventions—supportive-expressive peer social support groups and strengths-based case management—to determine their impact on health outcomes and quality of life among rural older PLH in the Southern U.S. If efficacious, these interventions could be disseminated by organizations serving rural PLH throughout the U.S. and result in improved health outcomes and quality of life in this population.

CRITIQUE 1

Significance: 1
Investigator(s): 1
Innovation: 1
Approach: 2
Environment: 2

Overall Impact: The case for supportive interventions for older PLWH who are from southern rural areas is well made by an appropriate and strong research team. The investigators have examined potential evidence-based support interventions in pilot studies and have identified two that show promise for improving both mental health and HIV management. Both interventions are offered remotely (by zoom or telephone). One intervention is individual-focused and addresses systems-level problem-solving. The other intervention is group focused and addresses socioemotional supports. The two interventions address previous pilot-study-identified barriers across socioecological model levels of influence. In this application the two interventions are tested both separately and together relative to a comparison group in a factorial design. As the interventions may be delivered separately or together in actual practice, it is important to know both their individual and synergistic effects. Methods for intervention delivery are manualized and a strong set of intervention delivery fidelity measures will be assessed. Barriers to be addressed in the interventions are clearly articulated and assessed by appropriate measures for which there is evidence of adequate internal reliability. Outcome measures are also clearly identified and assessed with strong measures. Viral load is measured both biologically and by self-report. Minor concerns about the trial are noted for further consideration but with little overall impact on enthusiasm for this application. More thought needs to be given to sexual orientation/gender identity and educational level both in regard to study design elements (randomization, analysis) as well as intervention (i.e., Intervention and intervention group composition).

Likewise, differences by face-to-face interaction via zoom and voice only interaction via telephone need more consideration. Finally, although problem-solving regarding systems-level HIV care concerns are addressed in the individual level intervention, the application does not clearly state that local HIV care resources will be identified in preparation both for responses in times of need for protections of human subjects as well as maximally helpful systems-level intervention problem-solving and advice. As these concerns can all be addressed with consideration and minor modifications and are not likely to change the essential elements of this application, they do not significantly decrease enthusiasm for this excellent application.

1. Significance:

Strengths

The researchers make a strong case that:

- Older southern rural persons with HIV are at high risk for many challenges regarding HIV care and other related physical and mental health problems.
- This study poses two virtual interventions with theoretically plausible beneficial impacts as well as preliminary evidence of their effectiveness.
- A trial of these interventions with the older southern rural persons with HIV is needed.
- The study interventions are appropriately framed around the socioecological model and used both individual and group-based intervention.

Weaknesses

- None noted by reviewer.

2. Investigator(s):

Strengths

- Excellent PI and team.
- The MPI plan is well justified and appropriate; the roles of the MPIs are well delineated, and they have developed protocols for communication and conflict resolution.

Weaknesses

- None noted by reviewer.

3. Innovation:

Strengths

- The interventions are delivered remotely (zoom and phone).
- Focus on older rural person's regarding HIV.

Weaknesses

- None noted by reviewer.

4. Approach:

Strengths

- The 2 x 2 factorial trial design is very strong.

- Strong preliminary studies.
- Strong measures with adequate reliability.
- Randomization to four conditions blocked by sex and race.
- SBCM intervention includes technology access goals.
- Use of biologic viral load measurement to compare to self-report.
- Interventions delivered through zoom, phone, and mailed materials.
- Strong fidelity measures.

Weaknesses

- Randomization only blocked by sex and race and not blocked by education level, sexual orientation which may also confound and be a factor related to interventions.
- Differences in intervention when delivered by phone, computer, or smartphone should be assessed.
- Confounding by whether the support group is mixed gender or single gender should be addressed in exploratory analysis.
- Regarding the implementation of interventions, if interventions are provided for mixed vs. single gender groups, then providing them for hetero vs. minority sexual orientation would also be appropriate.
- All groups should receive the mailed resources so that intervention effects are over and above the effect of this freely available, low-cost comparison effect.

5. Environment:

Strengths

- Good resources and environment.

Weaknesses

- It is important for the interventionists to have clear lists of available and acceptable referral points for needed services. While issues such as transportation, etc. are discussed as part of problem solving, it is not clear that interventionists will be able to offer critical resource advice in all study rural areas.

Study Timeline:

Strengths

- None noted by reviewer.

Weaknesses

- None identified.

Protections for Human Subjects:

Acceptable Risks and/or Adequate Protections.

- HIV care resources that can be accessed by participants need to be identified for the rural study areas so that participants in need can find necessary care.

Data and Safety Monitoring Plan (Applicable for Clinical Trials Only):
Acceptable

Inclusion Plans:

- Sex/Gender: Distribution justified scientifically.
- Race/Ethnicity: Distribution justified scientifically.
- For NIH-Defined Phase III trials, Plans for valid design and analysis: Scientifically acceptable.
- Inclusion/Exclusion Based on Age: Distribution justified scientifically.

Vertebrate Animals:
Not Applicable (No Vertebrate Animals)

Biohazards:
Acceptable

Applications from Foreign Organizations:
Not Applicable (No Foreign Organizations)

Select Agents:
Not Applicable (No Select Agents)

Resource Sharing Plans:
Acceptable

Authentication of Key Biological and/or Chemical Resources:
Not Applicable (No Relevant Resources)

Budget and Period of Support:
Recommend as Requested

CRITIQUE 2

Significance: 3
Investigator(s): 3
Innovation: 2
Approach: 5
Environment: 4

Overall Impact: There are some major strengths of this project, namely the focus on filling a major gap in intervention research with rural older people living with HIV. This study is poised to begin to address major urban-rural disparities in HIV care access as well. The study also has much promise as the team has taken a developmental approach and used pilot study and qualitative findings along with research to identify key intervention targets and plans to use evidence-based interventions for support groups and case management. Remote delivery of the interventions is novel and has high potential for reaching people in rural areas, although there may be limitations related to internet access and digital literacy. The team also proposes novel use of home-based dried blood spot collection, which moves the field forward in making viral load monitoring more accessible remotely. The team is quite strong, including a MPI team with complementary skills and experience, a Co-Investigator who leads work to address HIV in Southern States, another Co-Investigator committed to making the intervention relevant to the local contexts, and a Co-Investigator with expertise in digital interventions and home-based DBS methods. A weakness of the chosen interventions is the reliance on the resources and systems in place in rural areas to which participants can be referred. It is quite possible that there are not resources locally available to meet material needs and services critical to HIV care engagement like transportation, which can be surmised from the lack of pilot data showing change in service access and material needs. Outcomes may be most reliant on the resources available in the local communities where participants are recruited from, which will likely reduce impact of this proposal. The choice of a factorial design is limiting in terms of potential impact, especially given the major gap in interventions for rural people living with HIV. Overall, this is a strong proposal and team, but potential impact may be limited by the study design and structural barriers participants face.

1. Significance:

Strengths

- Developmental approach with strong preliminary efficacy data increases the chance of potential impact of this proposed research.
- This proposal is significant in addressing urban rural disparities in HIV care outcomes.
- Little to no intervention research is being conducted with rural older people living with HIV, thus this project fills an important gap in HIV research and services.

Weaknesses

- The social ecological model could be used to integrate a whole host of micro and macro influences from living in a rural area and being older that affect HIV care outcomes. The mechanisms of action in this proposal seem only loosely tied to theory.
- Rationale for the intervention components is not grounded well in the prior research conducted by the team. Directionality in the preliminary data they present are problematic as the team uses cross sectional data in which directionality is not clear. For example, it could be that being virally suppressed reduced feelings of HIV stigma, while the team presents data as showing HIV stigma increased viral suppression.
- Only one of the pilot aims (health related quality of life) was significantly and positively different between baseline and 3 month outcomes. And no significant differences were achieved in perhaps the most important outcomes of reducing structural barriers like transportation and materials hardship.
- This study design choice compromises maximum potential benefit for participants. The rationale for using the factorial design is not terribly strong, nor necessarily true (i.e., CBOs will not be able to implement both interventions). Two components in the team’s prior research were found

to be feasible and acceptable, suggesting investigators test both of these interventions together given the needs of rural older people living with HIV.

2. Investigator(s):

Strengths

- MPI plan clearly outlines roles of each MPI, their complementary contributions via medical and behavioral expertise and outline plans for addressing conflict.
- Inclusion of Dr. Quinn, who will bring a focus on connecting to local rural organizations across Southern states, is important given this proposal is not being led by local scientists.
- Existing collaboration with Dafina Ward from the Southern AIDS Coalition is a strength.
- Dr. Hirshfield's inclusion is a strength given her expertise in online interventions and home DBS collection.

Weaknesses

- Unclear what the role of Dr. John will be on this proposal, especially as most of this work is in prevention with sexual minority men in urban areas.
- Seems like there may be biostatistical overlap for Dr. Walsh and McAuliffe.

3. Innovation:

Strengths

- Remote delivery of intervention components is novel and well-tailored to meeting implementation challenges due to geographically dispersed regions and lack of availability of HIV care and support services in rural areas.
- Testing of home dried blood spot with this population is the ultimate proof of concept for wide adaptation and use.

Weaknesses

- None.

4. Approach:

Strengths

- Strong evidence that a diverse group of rural older people living with HIV in need of support to increase HIV care outcomes can be recruited using methods previously tested by the team.
- Real world preliminary efficacy (i.e., intervention offered without incentives) is a real strength pointing to high.

Weaknesses

- Qualitative data from first THRIVE seem to point to major structural barriers as main barriers to positive HIV care outcomes. These data call into question whether the services needed for rural people living with HIV care are present.
- Latinx people will be recruited but there is no information about Spanish capacity on the team. Missed opportunity not to include Spanish-speaking participants.

- Investigators mention that intervention delivery will be via Zoom or by phone. Data on zoom feasibility given need for considerable bandwidth and limited internet access in many rural areas along with digital literacy barriers mentioned in the significance is needed. For those forced to use phones, rapport and connection needed to most benefit from social support groups (connection, reduced isolation) and case management (depression) and make strides in HIV care engagement may be limited. At the least, these important implementation barriers and impacts should be assessed in exploratory analyses.
- Strengths-based case management seems better suited to settings where there are support services readily available. Empowerment to access resources is only useful in settings where resources are available. The expectation that PLH in rural areas will be able to find resources to meet such basic needs as transportation, food, internet access seems mismatched from the resources available in these areas and based on participant means as outlined by the study team.
- Investigators intend to recruit sexual minority men and racial/ethnic minority people. In addition to having groups by gender, it seems that specific content and organizing is needed for sexual and racial minority men who may face intersectional stigmas not experienced by others in the groups.

5. Environment:

Strengths

- The Medical College of Wisconsin and Center for AIDS Intervention Research has the research infrastructure and resources to complete the proposed research.
- The CFAR Basic Core lab has the capacity to analyze the remotely collected DBS.

Weaknesses

- Having a local site where services are available as a partner may be helpful for understanding the need (or lack thereof) for ancillary services to support adherence and to examine implementation of this intervention if there ends up being evidence efficacy.

Study Timeline:

Strengths

- Recruitment and enrollment targets seem feasible and are well described.

Weaknesses

- None.

Protections for Human Subjects:

Acceptable Risks and/or Adequate Protections.

Data and Safety Monitoring Plan (Applicable for Clinical Trials Only):

Acceptable

- DSMP proposed and no DSMB.

Inclusion Plans:

- Sex/Gender: Distribution justified scientifically.
- Race/Ethnicity: Distribution justified scientifically.
- For NIH-Defined Phase III trials, Plans for valid design and analysis: Not Applicable
- Inclusion/Exclusion Based on Age: Distribution justified scientifically.

Vertebrate Animals:
Not Applicable (No Vertebrate Animals)

Biohazards:
Acceptable

Applications from Foreign Organizations:
Not Applicable (No Foreign Organizations)

Select Agents:
Not Applicable (No Select Agents)

Resource Sharing Plans:
Acceptable

Authentication of Key Biological and/or Chemical Resources:
Acceptable

Budget and Period of Support:
Recommend as Requested

CRITIQUE 3

Significance: 1
Investigator(s): 1
Innovation: 2
Approach: 3
Environment: 1

Overall Impact: This is an excellent R01 application that proposes a clinical trial to investigate the efficacy of two different interventions – supportive-expressive peer social support groups vs. strengths-based case management interventions – in aging people living with HIV (PLWH) in rural US. Significance is extremely high. The investigators' team and research environment are outstanding and the focus on older PLWH in rural areas of USA is quite innovative. Enthusiasm for an otherwise high

impact proposal is only slightly dampened by minor weaknesses in the approach concerning potential number of enrolled patients and dropouts.

1. Significance:

Strengths

- USA rural areas harbor a significant number of PLWH, and HIV has become increasingly burdensome.
- PLWH in rural areas are less likely to remain immunosuppressed. Therefore, evaluating effective interventions in such a population is highly significant.
- Equally significant is the focus on the aging (>50 yo) HIV+ population, which presents additional challenges and is becoming larger in the US.
- The effectiveness of both interventions that will evaluated (supportive-expressive peer social support groups and strengths-based case management interventions) is supported by robust preliminary studies.

Weaknesses

- None noticed.

2. Investigator(s):

Strengths

- Strong MPIs with complementary expertise in psychology/HIV (Dr. Walsh) and infectious diseases/HIV (Dr. Petroll).
- The MPIs have a successful track record of collaboration through a previously awarded R56 and already co-authored several publications.
- The MPI plan is well outlined, including conflict resolution and contingencies in the unlikely case of change in PI location.

Weaknesses

- None noticed.

3. Innovation:

Strengths

- The focus on older PLWH in rural areas of USA is innovative.
- Another interesting innovation is the testing of remotely delivered interventions.

Weaknesses

- Factorial study design and use of dried blood spots for measuring VL is not very innovative.

4. Approach:

Strengths

- Recruitment strategies are clearly outlined and seem adequate to enroll the targeted number of participants.

- Interventions (8-weekly, 90-minutes support meetings), quality control, and fidelity monitoring are adequately discussed.
- The power analysis shows sufficient power to detect difference between the two interventions.

Weaknesses

- Although it seems likely that a sufficient number of participants will be enrolled, no alternatives are discussed in case such a number should not be reached.
- There is no discussion about participants that may drop out from the study or whether this might be a problem in rural and relatively isolated areas.

5. Environment:

Strengths

- The Medical College of Wisconsin and SUNY Downstate Medical Center are strong environments for the proposed research, as demonstrated by previous pilot trials carried out by the MPIs of the application.

Weaknesses

- None.

Study Timeline:

Strengths

- The timeline is clearly outlined in a table (page 105 of the application) detailing the different phases of the trial. Data collection will take place in Y1-Y3, while Y4 will be dedicated to manuscripts preparation and dissemination of results to community partners.

Weaknesses

- None noticed.

Protections for Human Subjects:

Acceptable Risks and/or Adequate Protections.

Data and Safety Monitoring Plan (Applicable for Clinical Trials Only):

Acceptable

Inclusion Plans:

- Sex/Gender: Distribution justified scientifically.
- Race/Ethnicity: Distribution justified scientifically.
- For NIH-Defined Phase III trials, Plans for valid design and analysis: Not Applicable
- Inclusion/Exclusion Based on Age: Distribution justified scientifically.

Vertebrate Animals:

Not Applicable (No Vertebrate Animals)

Biohazards:
Not Applicable (No Biohazards)

Applications from Foreign Organizations:
Not Applicable (No Foreign Organizations)

Select Agents:
Not Applicable (No Select Agents)

Resource Sharing Plans:
Acceptable

Authentication of Key Biological and/or Chemical Resources:
Not Applicable (No Relevant Resources)

Budget and Period of Support:
Recommend as Requested

THE FOLLOWING SECTIONS WERE PREPARED BY THE SCIENTIFIC REVIEW OFFICER TO SUMMARIZE THE OUTCOME OF DISCUSSIONS OF THE REVIEW COMMITTEE, OR REVIEWERS' WRITTEN CRITIQUES, ON THE FOLLOWING ISSUES:

PROTECTION OF HUMAN SUBJECTS: ACCEPTABLE

INCLUSION OF WOMEN PLAN: ACCEPTABLE

INCLUSION OF MINORITIES PLAN: ACCEPTABLE

INCLUSION ACROSS THE LIFESPAN: ACCEPTABLE

COMMITTEE BUDGET RECOMMENDATIONS: The budget was recommended as requested.

Footnotes for 1 R01 NR020770-01; PI Name: Walsh, Jennifer Lynn

NIH has modified its policy regarding the receipt of resubmissions (amended applications).See Guide Notice NOT-OD-18-197 at https://grants.nih.gov/grants/guide/notice-files/NOT-OD-18-197.html. The impact/priority score is calculated after discussion of an application by averaging the overall scores (1-9) given by all voting reviewers on the committee and multiplying by 10. The criterion scores are submitted prior to the meeting by the individual reviewers assigned to an application, and are not discussed specifically at the review meeting

or calculated into the overall impact score. Some applications also receive a percentile ranking. For details on the review process, see http://grants.nih.gov/grants/peer_review_process.htm#scoring.

MEETING ROSTER

Population and Public Health Approaches to HIV/AIDS Study Section
Healthcare Delivery and Methodologies Integrated Review Group
CENTER FOR SCIENTIFIC REVIEW
PPAH
07/14/2022 - 07/15/2022

Notice of NIH Policy to All Applicants: Meeting rosters are provided for information purposes only. Applicant investigators and institutional officials must not communicate directly with study section members about an application before or after the review. Failure to observe this policy will create a serious breach of integrity in the peer review process, and may lead to actions outlined in NOT-OD-22-044 at https://grants.nih.gov/grants/guide/notice-files/NOT-OD-22-044.html, including removal of the application from immediate review.

CHAIRPERSON(S)

YOUNG, APRIL MARIE, MPH, PHD
ASSOCIATE PROFESSOR
DEPARTMENT OF EPIDEMIOLOGY
COLLEGE OF PUBLIC HEALTH
UNIVERSITY OF KENTUCKY
LEXINGTON, KY 40536

MEMBERS

ABUOGI, LISA LYNN, MD
ASSOCIATE PROFESSOR
DEPARTMENT OF PEDIATRICS
SCHOOL OF MEDICINE
UNIVERSITY OF COLORADO, DENVER
AURORA, CO 80045

AMIRKHANIAN, YURI A, PHD
PROFESSOR
DEPARTMENT OF PSYCHIATRY AND BEHAVIORAL MEDICINE
CENTER FOR AIDS INTERVENTION RESEARCH
MEDICAL COLLEGE OF WISCONSIN
MILWAUKEE, WI 53202

ARONSON, IAN DAVID, PHD *
ASSOCIATE RESEARCH SCIENTIST
CENTER FOR DRUG USE AND HIV HCV RESEARCH
SCHOOL OF GLOBAL PUBLIC HEALTH
NEW YORK UNIVERSITY
NEW YORK, NY 10003

BARNIGHAUSEN, TILL, MD
PROFESSOR AND DIRECTOR
HEIDELBERG INSTITUTE OF GLOBAL HEALTH
UNIVERSITY OF HEIDELBERG
HEIDLEBURG, GERMANY 69120
GERMANY

BLANK, MICHAEL B, PHD *
PROFESSOR
DEPARTMENT OF PSYCHIATRY
PERELMAN SCHOOL OF MEDICINE
UNIVERSITY OF PENNSYLVANIA
PHILADELPHIA, PA 19104

BOEKELOO, BRADLEY O, PHD *
PROFESSOR
DEPARTMENT OF BEHAVIORAL AND COMMUNITY HEALTH
SCHOOL OF PUBLIC HEALTH
UNIVERSITY OF MARYLAND
COLLEGE PARK, MD 20742

BUDHWANI, HENNA, PHD *
ASSOCIATE PROFESSOR
DEPARTMENT OF HEALTH CARE ORGANIZATION
AND POLICY
SCHOOL OF PUBLIC HEALTH
THE UNIVERSITY OF ALABAMA AT BIRMINGHAM
BIRMINGHAM, AL 35924

DARBES, LYNAE A, PHD
ASSOCIATE PROFESSOR
DEPARTMENT OF HEALTH BEHAVIOR
AND BIOLOGICAL SCIENCES
SCHOOL OF NURSING
UNIVERSITY OF MICHIGAN
ANN ARBOR, MI 48109

DEGRUTTOLA, VICTOR GERARD, DSC *
PROFESSOR
DEPARTMENT OF BIOSTATISTICS
SCHOOL OF PUBLIC HEALTH
HARVARD UNIVERSITY
BOSTON, MA 02115

EDLAND, STEVEN DYAL, PHD *
PROFESSOR
DEPARTMENT OF FAMILY MEDICINE AND
PUBLIC HEALTH
UNIVERSITY OF CALIFORNIA, SAN DIEGO
LA JOLLA, CA 92093

FOX, MATTHEW ALEXANDER PEASE, DSC, MPH *
PROFESSOR
DEPARTMENT OF EPIDEMIOLOGY
SCHOOL OF PUBLIC HEALTH
BOSTON UNIVERSITY
BOSTON, MA 02118

FUJIMOTO, KAYO, PHD
DISTINGUISHED PROFESSOR
DEPARTMENT OF HEALTH PROMOTION
AND BEHAVIORAL SCIENCES
SCHOOL OF PUBLIC HEALTH
UNIVERSITY OF TEXAS HEALTH SCIENCE CENTER
HOUSTON, TX 77030

GOEDEL, WILLIAM C, PHD *
ASSISTANT PROFESSOR
DEPARTMENT OF EPIDEMIOLOGY
SCHOOL OF PUBLIC HEALTH
BROWN UNIVERSITY
PROVIDENCE, RI 02912

GOPALAPPA, CHAITRA, PHD *
ASSOCIATE PROFESSOR
DEPARTMENT OF MECHANICAL
AND INDUSTRIAL ENGINEERING
COLLEGE OF ENGINEERING
UNIVERSITY OF MASSACHUSETTS, AMHERST
AMHERST, MA 01003

HAHN, JUDITH ALISSA, PHD *
PROFESSOR
DEPARTMENT OF MEDICINE
SAN FRANCISCO GENERAL HOSPITAL
UNIVERSITY OF CALIFORNIA, SAN FRANCISCO
SAN FRANCISCO, CA 94143

HECKMAN, TIMOTHY GLENN, PHD *
PROFESSOR AND ASSOCIATE DEAN
DEPARTMENT OF HEALTH PROMOTION AND BEHAVIOR
COLLEGE OF PUBLIC HEALTH
UNIVERSITY OF GEORGIA
ATHENS, GA 30602

HERBECK, JOSHUA T, PHD *
ASSISTANT PROFESSOR
DEPARTMENT OF GLOBAL HEALTH
UNIVERSITY OF WASHINGTON
SEATTLE, WA 98195

JENNESS, SAMUEL, MPH, PHD *
ASSOCIATE PROFESSOR
DEPARTMENT OF EPIDEMIOLOGY
ROLLINS SCHOOL OF PUBLIC HEALTH
EMORY UNIVERSITY
ATLANTA, GA 30030

KERSHAW, TRACE S, PHD *
PROFESSOR
CENTER FOR INTERDISCIPLINARY RESEARCH ON AIDS
DEPARTMENT OF EPIDEMIOLOGY
SCHOOL OF PUBLIC HEALTH
YALE UNIVERSITY
NEW HAVEN, CT 06510

LAU, BRYAN, PHD *
PROFESSOR
DEPARTMENT OF EPIDEMIOLOGY
BLOOMBERG SCHOOL OF PUBLIC HEALTH
JOHNS HOPKINS UNIVERSITY SCHOOL OF MEDICINE
BALTIMORE, MD 21205

LEITNER, THOMAS K, PHD *
STAFF SCIENTIST
THEORETICAL BIOLOGY AND BIOPHYSICS GROUP
LOS ALAMOS NATIONAL LABORATORY
LOS ALAMOS, NM 87545

MCMAHON, JAMES M, PHD
ASSOCIATE PROFESSOR AND ENDOWED CHAIR
SCHOOL OF NURSING
UNIVERSITY OF ROCHESTER MEDICAL CENTER
ROCHESTER, NY 14642

MEEK, ERIN, DRPH, MPH *
SENIOR RESEARCH SCIENTIST
AIDS OFFICE
SAN FRANCISCO DEPARTMENT OF PUBLIC HEALTH
SAN FRANCISCO, CA 94102

NASH, DENIS, MPH, PHD *
PROFESSOR
DEPARTMENT OF EPIDEMIOLOGY AND BIOSTATISTICS
SCHOOL OF PUBLIC HEALTH
CITY UNIVERSITY OF NEW YORK
NEW YORK, NY 10035

NIJHAWAN, ANK ELISABETH, MD, MPH *
ASSOCIATE PROFESSOR
INTERNAL MEDICINE, DIVISION OF INFECTIOUS DISEASES
UT SOUTHWESTERN MEDICAL CENTER
DALLAS, TX 75390

OUTLAW, ANGULIQUE Y, PHD *
ASSOCIATE PROFESSOR
DEPARTMENT OF FAMILY MEDICINE AND
PUBLIC HEALTH SCIENCES
SCHOOL OF MEDICINE
WAYNE STATE UNIVERSITY
DETROIT, MI 48202

PHO, MAI TUYET, MD, MPH
ASSOCIATE PROFESSOR
DEPARTMENT OF MEDICINE
SECTION OF INFECTIOUS DISEASES AND GLOBAL HEALTH
UNIVERSITY OF CHICAGO MEDICAL CENTER
CHICAGO, IL 60637

RAMIREZ (KITCHEN), CHRISTINA MICHELLE, PHD *
ASSOCIATE PROFESSOR
DEPARTMENT OF BIOSTATISTICS
SCHOOL OF PUBLIC HEALTH
UNIVERSITY OF CALIFORNIA, LOS ANGELES
LOS ANGELES, CA 90095

SALEEM, HANEEFA TASLEEM, MPH, PHD *
ASSISTANT PROFESSOR
BLOOMBERG SCHOOL OF PUBLIC HEATH
JOHNS HOPKINS UNIVERSITY
BALTIMORE, MD 21205

SALEMI, MARCO, PHD
PROFESSOR
DEPARTMENT OF PATHOLOGY, IMMUNOLOGY,
AND LABORATORY MEDICINE
COLLEGE OF MEDICINE
UNIVERSITY OF FLORIDA
GAINESVILLE, FL 32610

SEAL, DAVID W, PHD *
PROFESSOR
DEPARTMENT OF GLOBAL COMMUNITY HEALTH
AND BEHAVIORAL SCIENCES
SCHOOL OF PUBLIC HEALTH AND TROPICAL MEDICINE
TULANE UNIVERSITY
NEW ORLEANS, LA 70112

VARDAVAS, RAFFAELE, PHD *
MATHEMATICIAN
FACULTY PARDEE RAND GRADUATE SCHOOL
RAND CORPORATION
SANTA MONICA, CA 90407

WITTE, SUSAN S, PHD
PROFESSOR
SCHOOL OF SOCIAL WORK
COLUMBIA UNIVERSITY
NEW YORK, NY 10027

MAIL REVIEWER(S)

GOLIN, CAROL E, MD
PROFESSOR
DEPARTMENT OF HEALTH BEHAVIOR AND
HEALTH EDUCATION
UNIVERSITY OF NORTH CAROLINA
CHAPEL HILL, NC 27599

SCIENTIFIC REVIEW OFFICER

GUERRIER, JOSE H, PHD
SCIENTIFIC REVIEW OFFICER
CENTER FOR SCIENTIFIC REVIEW
NATIONAL INSTITUTES OF HEALTH
BETHESDA, MD 20892

* Temporary Member. For grant applications, temporary members may participate in the entire meeting or may review only selected applications as needed.

Consultants are required to absent themselves from the room during the review of any application if their presence would constitute or appear to constitute a conflict of interest.
